# Perinatal iron deficiency and neurocognitive development

**DOI:** 10.3389/fnhum.2013.00585

**Published:** 2013-09-23

**Authors:** Emily C. Radlowski, Rodney W. Johnson

**Affiliations:** ^1^Department of Animal Sciences, University of IllinoisUrbana, IL, USA; ^2^Division of Nutritional Sciences, University of IllinoisUrbana, IL, USA; ^3^Neuroscience Program, University of IllinoisUrbana, IL, USA

**Keywords:** nutrition, iron deficiency, cognitive development, learning, memory, hippocampus

## Abstract

Iron deficiency is the most common form of nutrient deficiency worldwide. It is highly prevalent due to the limited availability of high quality food in developing countries and poor dietary habits in industrialized countries. According to the World Health Organization, it affects nearly 2 billion people and up to 50% of women who are pregnant. Maternal anemia during pregnancy is especially burdensome to healthy neurodevelopment in the fetus because iron is needed for proper neurogenesis, development, and myelination. Maternal anemia also increases the risk of low birth weight, either due to premature birth or fetal growth restriction, which is associated with delayed neurocognitive development and even psychiatric illness. As rapid neurodevelopment continues after birth infants that received sufficient iron *in utero*, but that receive a low iron diet after 6 months of age, also show deficits in neurocognitive development, including impairments in learning and memory. Unfortunately, the neurocognitive complications of iron deficiency during critical pre- and postnatal periods of brain development are difficult to remedy, persisting into adulthood. Thus, preventing iron deficiency in the pre- and postnatal periods is critical as is devising new means to recapture cognitive function in individuals who experienced early iron deficiency. This review will discuss the prevalence of pre- and postnatal iron deficiency, the mechanism, and effects of iron deficiency on brain and cognitive development.

## Introduction

Iron deficiency is the primary cause of anemia, which affects roughly one-quarter of the world’s population (McLean et al., 2009). As the most prevalent micronutrient deficiency in the world, iron deficiency affects all age groups, with the most common being children between the ages of 0 and 5 years (McLean et al., 2009). It was previously thought that neonates were protected from iron deficiency, in all but the most severe cases of maternal anemia, due to mobilization of iron stores accumulated *in utero* (Allen, 2000). However, it is now documented that even mild iron deficiency in the mother reduces iron stores in the fetus, resulting in a neonatal iron-deficient condition (Rao and Georgieff, 2002). Neonatal iron deficiency is also greater in infants born prematurely or of a diabetic mother (Lozoff et al., 2006). As the incidence of both these conditions is increasing worldwide (Beck et al., 2010; Martin et al., 2010; Danaei et al., 2011), it is likely that iron deficiency in infants will become an even greater concern in the future.

The impact of perinatal iron deficiency on human brain and cognitive development is of particular interest because the brain growth spurt that begins in the last one-third of pregnancy continues the first 2 years after birth due to dendritic growth, synaptogenesis, and glial cell proliferation (Courchesne et al., 2000; Gogtay et al., 2006). Neurogenesis in the hippocampal dentate gyrus also persists in the neonatal period (and throughout adulthood), and recent evidence indicates lingering neurogenesis in different cortical regions, including the prefrontal cortex in human infants (Sanai et al., 2011; Feliciano and Bordey, 2012). Total brain volume doubles the first year and reaches 80–90% of adult volume by age two (Knickmeyer et al., 2008). This phase of rapid growth represents a sensitive period, wherein environmental insults alter neurodevelopment (McEwen, 1999).

One such insult is iron deficiency. The hippocampus, a brain area important in learning, memory, and cognition, is highly susceptible to iron deficiency during the late fetal and early neonatal time period. For example, iron deficiency in the perinatal period is associated with altered expression of genes critical for hippocampal development and function (Carlson et al., 2007). Moreover, early iron deficiency causes neurocognitive dysfunction both during deficiency and after repletion (Beard and Connor, 2003; Jorgenson et al., 2005). Thus, preventing iron deficiency in the perinatal period is critical as is devising new means to recapture cognitive function in individuals who experienced early iron deficiency. Although iron deficiency is a major concern across the lifespan, this review will focus on the prevalence of pre- and postnatal iron deficiency, perinatal iron homeostasis, and the effects of perinatal iron deficiency on brain and cognitive development.

## Prevalence of iron deficiency and iron deficiency anemia

Of all micronutrient deficiencies, iron deficiency is most prevalent worldwide. It affects all age groups and demographics; however, prevalence is higher in pubescent women, pregnant women, infants and preschool-age children. Rates of anemia in non-pregnant women of childbearing age reach approximately 40% in developing countries and 20% in industrialized countries (McLean et al., 2009). The incidence of iron deficiency anemia increases further during pregnancy, with rates in developing and industrialized countries reaching 59% and 24%, respectively. The large increase in iron deficiency in pregnant women in developing countries may be due to poor nutrition education and lack of iron supplements (Pasricha et al., 2013).

Infants born full term with an appropriate weight for gestation have iron stores that are adequate for about 6 months. This is vital because the neonatal gut is developmentally immature and infants are unable to regulate iron absorption until 6–9 months of age (Domellöf et al., 2002). Furthermore, iron content of breast milk is very low (approximately 35–40 μg/dL). Although information on iron deficiency in infants less than 1 year of age is lacking, the Pediatric Nutrition Surveillance System (PedNSS) reported hemoglobin in a national sample of infants from families participating in the Special Supplemental Nutrition Program for Women, Infants, and Children (i.e., Supplemental Nutrition Assistance Program (SNAP; Polhamus et al., 2004)). In 2003, 16.2% of infants aged 6–11 months, and 15% of children aged 12–17 months qualified as having iron deficiency anemia (Polhamus et al., 2004). In a Brazilian community, iron deficiency anemia in infants (average age 11.6 months) reached nearly 26% (Konstantyner et al., 2012); and in Canada, the rate of iron deficiency anemia in Cree Indian infants (average age 9 months) was nearly 32% (Willows et al., 2000). Sadly, in most countries including the U.S., the occurrence of iron deficiency worsens in preschool-aged children (CDC, 2011; see Table 1).

**Table 1:** **Prevalence of iron deficiency anemia around the world**.

		**Iron deficiency anemia**	**Iron deficiency**
**Industrialized countries**	**Pregnant women**	24% *	Iron deficiency is not commonly tested for and rates around the world are unknown at this time, but estimated to be much higher than for iron deficiency anemia
	**Infant (0–12 months)**	16.2% ^†^	
	**Children (1–5 years)**	25% *	
**Developing countries**	**Pregnant women**	59% *	
	**Infant (0–12 months)**	40% ^‡^	
	**Children (1–5 years)**	67% *	

* *McLean et al. (2009)*.

^†^ *Polhamus et al. (2004)*.

^‡^ *Chaparro (2008)*.

### Maternal iron deficiency and iron deficiency anemia: effects on neonatal iron status

As iron is concerned, the developing fetus was once considered a “perfect parasite,” able to acquire sufficient iron from the mother even when she was iron deficient (Young et al., 2012). This notion has fallen by the wayside, however, and it is now clear that neonatal iron stores can be compromised when the mother is iron deficient or anemic. For example, under steady-state conditions serum ferritin concentration correlates with total body iron stores. New born infants from iron deficient mothers with low serum ferritin levels also had low serum ferritin indicating there is a limited capacity for the fetus to accumulate iron from low maternal stores (Jaime-Perez et al., 2005). Gestational iron deficiency also appears to have a programming effect on the physiologic mechanisms responsible for iron homeostasis resulting in offspring that are more likely to develop iron deficiency in the future regardless of adequate nutrition (De Pee et al., 2002; Georgieff et al., 2002; Emamghorashi and Heidari, 2004. Multiple studies have shown that iron status in infants born to iron deficient mothers is still abnormally low 9 months after birth despite being provided adequate dietary iron (Georgieff et al., 2002; Geltman et al., 2004).

The timing of iron deficiency during pregnancy is critical. First, there is an important need for iron early in pregnancy for neural development. In a recent study in rats, four dietary-feeding regimens were used to render the developing fetuses iron deficient at different stages of gestation. Maternal iron restriction beginning prior to conception and during the first one-third of pregnancy was associated with embryonic iron deficiency, postnatal anemia, reduced iron levels in the central nervous system, and decreased neural conduction velocities in an auditory brainstem response test conducted at postnatal day 45 (Mihaila et al., 2011). Importantly, the functional neural impairments were not induced when maternal iron restriction was initiated at the beginning of the last one-third of pregnancy. It’s also noteworthy that in this study, the mothers were not anemic; indicating poorly timed iron deficiency is sufficient to disrupt neural development and function. Second, as the fetal liver continues to grow, most (> 66%) of the infants total body iron is acquired during the third trimester of pregnancy (Allen, 2000). Hence, infants born preterm and/or low birth weight have poorer iron stores and are a high risk population for iron deficiency (Scholl, 2011).

### Iron homeostasis in the brain of infants

After 6 months of age, the blood-brain barrier is a major control point for the entry of iron into the brain, although the choroid plexus (the vasculature responsible for producing cerebrospinal fluid) is also a location of regulation for iron entry (Beard and Connor, 2003). The blood-brain barrier is important, as it prevents the brain from having direct access to the iron in the blood plasma, allowing for greater regulation (Piñero and Connor, 2000). Transferrin (Tf) receptors are present on the endothelial cells that make up the blood-brain barrier to allow for the binding and endocytosis of transferrin-bound iron into the brain (Beard, 2001). The rate of iron entry into the brain is increased during iron deficiency (Taylor et al., 1991), which is due to an increased amount of Tf receptors present on the cells of the blood-brain barrier (van Gelder et al., 1998), as well as a possible role for regulation by astrocytes (Beard and Connor, 2003).

In the newborn infant, the story is a bit different. At birth, the blood-brain barrier is incompletely developed: it prevents iron transport proteins from diffusing into the brain but it lacks the ability to transfer iron from the blood into the brain parenchyma (Collard, 2009). Studies done in rat pups showed that iron regulatory proteins, which are involved with the regulation of Tf receptor, were not readily expressed until post-natal day 15, when peak myelination occurs in the rat (Siddappa et al., 2002). It is not known for human infants when the blood-brain barrier fully matures, but it has been estimated to occur by 6 months of age (Rice and Barone, 2000). During prenatal development iron accumulates in the brain so levels are highest immediately after birth (Siddappa et al., 2003). Due to poor bioavailability and low levels of iron in human breast milk, infant brain iron concentration decreases the first 6 months after birth, until the gastrointestinal tract and blood-brain barrier mature and are able to absorb dietary iron and regulate its entry into the brain, respectively. This coincides with the onset of myelination and an increase in transferring mRNA levels in brain (Connor et al., 1987; Connor and Menzies, 1996; Roncagliolo et al., 1998). Since oligodendrocytes synthesize Tf, the brain is the only organ in which Tf mRNA increases post-natally (Bloch et al., 1985). The developmental timeline for mechanisms responsible for iron homeostasis underscore the importance of maternal iron status during pregnancy.

Once iron penetrates the blood-brain barrier, much less is known about how it is distributed to different brain regions. Most likely Tf (secreted by the choroid plexus, which is another point of regulation) is the primary method for the distribution of iron in the brain (Beard, 2001); however, it is also possible for iron to be transported by binding to ferritin (Piñero and Connor, 2000). The iron can then be taken up by cells in various brain regions that express receptors for Tf and/or ferritin, with the expression of these receptors as the mechanism of regulation (Beard and Connor, 2003). This leads to an uneven distribution of iron between the various brain regions, which changes throughout the lifetime (Beard, 2001). In general, the globus pallidus, red nucleus, substantia nigra, and caudate putamen have higher iron concentrations throughout life, and the brain tends to accumulate iron with age (Piñero and Connor, 2000).

### Neurocognitive deficits resulting from perinatal iron deficiency

One of the first studies to look at the effect of iron deficiency on cognition in humans used the Bayley Scales of Infant Development, which assess motor, language, and cognitive development in infants and toddlers, and compared 9–26 month old infants who were given iron supplements to those given a placebo. Each group was tested and then re-tested within 8 days of the initial exam. The results showed an improved scores within the Mental Development Index for the iron supplemented group (Oski and Honig, 1978), which resulted in a surge of interest in this topic (Yehuda et al., 2010). Many different studies have looked at iron deficiency during various times of development; however, the most sensitive period (and the period that can cause the most irreversible damage) is the neonatal period, which is between 0 and 24 months of age (Pollitt, 1993). Although supplementation with iron has been shown to correct some of the cognitive deficits during this period, lower I.Q. and achievement test scores have still been found after treatment (Lozoff, 1989).

Many studies have investigated the impact of neonatal iron status at various times during this critical period and the effects that decreased iron levels have on cognition. In Papua New Guinea, infants who were given an iron dextran shot at 2 months of age had longer attention spans at 1 year of age when compared to control (Heywood et al., 1989). A study done in Costa Rica found that infants who had lower iron levels scored lower in the Bayley Scales of Infant Development tests in both cognitive and motor skill tests (Lozoff, 1989). In Guatemala, researchers found that an intramuscular injection of iron dextran improved Bayley Scales of Infant Development test scores in babies 6–24 months old after just one week, while oral supplementation did not show an effect within the week studied (Lozoff et al., 1982). In Chile, infants 3 months of age, were given either iron fortified formula or control diet for 12 months (Walter et al., 1983). At the conclusion of the diet intervention, Bayley Scales of Infant Development were administered. After the initial test, all infants received a trial of orally administered ferrous sulfate daily and then retested within 15 days (Walter et al., 1983). A similar relationship between low iron levels and lower Bayley Scales of Infant Development scores was found (Walter et al., 1983). Studies in Indonesia found that 8 weeks of oral supplementation of iron in anemic preschool aged children reduced deficits in visual attention and concept acquisition compared to children who were not given supplementation (Soewondo et al., 1989). Also in Indonesia, when 12–18 month old infants that were diagnosed with iron deficiency anemia were given an oral iron intervention, Bayley Scales of Infant Development scores significantly improved compared to those given a placebo, even after only 4 months of supplementation (Idjradinata and Pollitt, 1993). Nine and twelve month old infants were tested for their ability to discriminate a highly familiar stimulus, their mother’s face, from a stranger’s face using an electroencephalogram (Burden et al., 2007). At 9 months infants that were iron sufficient showed greater attentional response to the mother’s face and greater updating of memory to the stranger’s face, while iron deficient infants did not show this response until 12 months of age, suggesting a delay in cognitive development (Burden et al., 2007). It has also been demonstrated that infants with low serum ferritin concentrations have abnormal auditory recognition memory (Siddappa et al., 2004). The infants in this study did not discriminate a familiar stimulus (mother’s voice) from a novel stimulus (stranger’s voice) with the same vehemence as an iron sufficient infant (Siddappa et al., 2004). These findings suggest abnormalities in structures that mediate recognition and memory function, including the hippocampus (Georgieff, 2008).

Being iron deficient at birth seems to cause long term deficits as well. Five year old children, who were born either iron deficient, scored lower on tests of language ability, fine-motor skills, and tractability, when compared to children who were iron sufficient at birth (Tamura et al., 2002). In Israel, it was found that children who were born premature and had low ferritin levels at birth performed significantly worse on tests involving spatial cognition and processing of auditory signals when tested at 9 to 10 years of age, even though their hemoglobin levels had returned to normal (Armony-Sivan et al., 2004; Yehuda and Yehuda, 2006). Another study showed similar results in that Costa Rican teens that were severely iron deficient during infancy, despite resolution of anemia while an infant, showed deficits when given neurocognitive tests (Trail Making test, Intra-Extra-dimensional Shift, Stockings of Cambridge, Spatial Working Memory, Rapid Visual Information Processing, Pattern Recognition Memory, and Spatial Recognition Memory) at the age of 19 years old, when compared to teens that were iron sufficient during infancy (Lukowski et al., 2010).

Of course, studies in animals have greatly advanced the knowledge of iron deficiency and its effects on cognitive performance. The fact that the avoidance response is affected by iron deficiency was one of the first cognitive aspects studied in rodents. Rats placed on an iron deficient diet at an early age showed deficits in both passive avoidance, where the rat had to inhibit its activity (i.e., not leave a platform and enter a chamber) in order to avoid shock, and active avoidance (i.e., where rats had to move into another chamber of the testing area in order to avoid shock) (Weinberg et al., 1979, 1981). Fear conditioning (as measured by heart rate deceleration in a cage where the rat had been shocked previously, as well as exposure to a tone that was played during the shock in a novel cage) was also impaired in iron deficient rats (McEchron et al., 2005).

Iron deficient diets also resulted in cognitive deficits in the Morris Water Maze, a hippocampal-dependent task which requires the rat to develop a spatial map of the area surrounding the pool in order to reach a hidden platform (Yehuda and Youdim, 1989). Similar deficits were observed in a water Y-maze (where the rat had two choices and a dry platform was placed at the end of the arm of the correct choice as a reward), including increased incorrect arm entries and a longer time to reach the platform (Yehuda et al., 1986), both of which indicate cognitive deficits. A recent study with piglets also demonstrated the effects of early-life iron deficiency on hippocampal-dependent learning (Rytych et al., 2012). Neonatal piglets placed on liquid diets of varying iron content (adequate, mildly deficient, and a severely deficient) 2 days after birth underwent repeated cognitive testing beginning about 2-weeks later using a T-maze task to measure spatial learning and memory. Severely iron deficient piglets were not able to successfully learn the task, while mildly deficient piglets took longer to learn compared to controls. In addition to poor performance, both sets of iron deficient piglets had less iron present in the hippocampus compared to controls (Rytych et al., 2012). These rodent and piglet studies confirmed that the magnitude of the cognitive effects observed correlated with the duration and severity of the iron deficiency.

The timing of iron deficiency during the perinatal period can also affect learning and memory. A pre-natal, post-natal, and pre+post-natal iron deficiency paradigm was used with mice pups to examine the effects of iron deficiency on learning and memory (Ranade et al., 2013). Mice pups that were pre-natal iron deficient or pre+ post-natal iron deficient performed poorly in a radial arm maze task; performance was better for pups in the post-natal deficient group, although their performance did not match that of pups provided adequate iron in both the pre- and postnatal periods. Even when pre-natal iron deficient pups became iron sufficient after birth, they still exhibited a poor ability to utilize reference memory, suggesting the hippocampus is highly vulnerable to iron deficiency in the pre-natal period (Ranade et al., 2013). However, the fact that performance of the post-natal iron deficient group was intermediate to the other iron deficient groups and control confirms that the window of vulnerability to iron deficiency for the hippocampus does not close immediately at birth.

Iron deficiency decreases brain iron concentration, which leads to numerous behavioral symptoms, such as irritability, apathy, reduced ability to concentrate, and other cognitive deficits (Piñero and Connor, 2000) (Table 2). Other important behavioral problems have been reported in association with iron deficiency as well. For example, children with Attention Deficit Hyperactivity Disorder (ADHD) were found to have lower levels of serum ferritin, an indication of reduced iron storage (Konofal et al., 2004). Iron-deficient animals develop ADHD-like behavior that has been linked to the dopaminergic system (Lahat et al., 2011). Deficits in motor development are also a symptom of iron deficiency in the neonate. In addition to cognitive development, the Bayley Scales of Infant Development also assesses fine and gross motor skills. Numerous studies (Lozoff et al., 1982; Walter et al., 1983; Lozoff, 1989; Idjradinata and Pollitt, 1993) indicate iron deficiency is associated with poorer scores in the motor function assessment within of the Bayley Scales of Infant Development. Further, anemic infants had low Mental and Psychomotor Development Index scores, even after 3 months of iron therapy. The anemic infants also showed deficits in language capability and coordination (Walter et al., 1989). In rodents, iron deficiency is associated with delayed development of surface righting, bar hold, forelimb placing, and negative geotaxis (Beard et al., 2006).

**Table 2:** **Neurobehavioral consequences of iron deficiency**.

**Affected process**	**Population**	**Measure**	**Iron supplement?**	**Supplement administration and timing**	**Result**	**Possible cause**	**Source**
**Behavior**	Costa Rican infants 12–23 months, IDA (*n* = 52), IS (*n* = 139)	Observation in clinic and at home using bayley scales of infant development. Spatial relations, affective state, behavior in relation to toys and in relation to caregiver	Yes	Behavior assessed before and after 3 months of iron therapy	Marked differences in behavior were found between the IDA and IS group despite resolution of anemia from iron therapy. IDA infants remained close to caregivers at all times, and showed increased fearfulness, wariness, hesitance, unhappiness, and tension	Inhibited neurotransmitter function, hypomyelination, and delayed neuromaturation from ID may lead to behavioral differences when compared to IS infants	Lozoff et al., 1998
**Behavior-ADHD**	French children, age 4–15 years old, ADHD (*n* = 45), control (*n* = 27)	Conners’ Parent Rating Scale (CPRS). Serum ferritin, hemoglobin, hematocrit, and iron levels in blood were measured	No	NA	Serum ferritin was significantly lower in children with ADHD compared to control, while other blood measure were the same. Serum ferritin levels inversely correlated with CPRS scores	Low ferritin may be responsible for altered dopaminergic neurotransmission which can affect brain dopaminergic activity in children and contribute to ADHD	Konofal et al., 2004
**Motor development**	Inner city infants, 9–10 months, ID (*n* = 28), IDA (*n* = 28), IS (*n* = 21)	Peabody Developmental Motor Scales, Infant Neurological International Battery (IFANIB), toy retrieval task	No	NA	Poorer motor function found in IDA and ID infants, compared to IS. 34% of IS infants were standing alone, if not walking (19%) by 9 months age, while only 19% of ID and IDA infants could stand alone. ID infants showed deficits in toy retrieval task as well	Impaired myelination in the corticospinal tract may delay/alter normal development and refinement of motor skills. ID induced changes to dopamine function within the basal ganglia may explain poor performance in toy retrieval task	Shafir et al., 2008
**Sensory systems**	Chilean children, ~4 years old, IDA (*n* = 41), IS (*n* = 43)	Auditory Brainstem Response (ABR), and Visual Evoked Potentials (VEP)	Yes	Children were supplemented for 6 months to a year when 6 month–18 months old. These are formerly deficient children	Formerly IDA children had significantly longer latencies for all ABR and VEP waves compared to IS children. Amplitudes were not different between groups	ID effects pathway transmission in both visual and auditory systems. May be due to hypomyelination. Differences in latency, but not amplitude, support this hypothesis. Latency changes relate to increases in conduction velocity during axonal myelination	Algarín et al., 2003
**IQ**	Egyptian children, age 6–12, IDA (*n* = 22), and IS (*n* = 16)	Weschler intelligence scale. Revised behavior problem checklist: conduct disorder, socialized aggression, attention problem-immaturity, anxiety-withdrawal, psychotic behavior, motor excess	No	NA	The mean IQ of the IDA group was significantly lower than the IS group. IDA children showed significant differences in motor control compared to the two other groups, and attention problems were higher in both anemic groups compared to IS, but highest in IDA	Iron deficiency can cause changes to hemoglobin concentrations, and mean corpuscular volume. In this study, both measures were predictive of attention deficit or motor excess	Mubarak et al., 2010
**Memory**	Chilean children, 10 years old, Formerly Iron Deficient (FID; *n* = 19) and control (*n* = 23)	Recognition memory task using Electrophysiological Recording and Processing (ERP)	Yes	Children were supplemented for 6 months to a year when 6 month–18 months old. These are formerly deficient children	Although accuracy within the task was the same for both groups, FID children took significantly longer to complete the task compared to controls. The FID group also had longer latency in the FN400 and P300 components of ERP testing, suggesting a delay in crucial memory searching processes and neural circuitry, respectively	Iron deficiency during formative years may have long lasting effects and cause deficiencies in neural circuitry and hypomyelination. The differences seen in the FID group in the FN400 component measure may also be due to difficulty accessing semantic memory to complete the task at the same level as the controls	Congdon et al., 2012

Abbreviations *ID* *Iron Deficiency* *IDA* *Iron Deficiency Anemia* *IS* *Iron Sufficient*

### Iron utilization within the brain

So what are some potential mechanisms underlying iron deficiency-related deficits in cognition? Iron is important for erythropoiesis, formation of hemoglobin and myoglobin, gene transcription, cellular enzyme reactions, and important oxidation-reduction actions (Lieu et al., 2001). All of these, of course, are important for proper brain function, so it is not surprising that iron deficiency results in behavioral disorders and deficits in learning and memory (Rao and Georgieff, 2007). In humans, the hippocampus matures most rapidly over a short period of time: from late gestation to 2–3 years of age. During this period, there is an increase in iron uptake and utilization as well as neurogenesis, dendrite growth, myelination, synaptogenesis, and neurotransmitter synthesis (Fretham et al., 2011). Hippocampal-dependent memory appears and matures between 3–18 months of age (Nelson, 1995). Because infants are unable to fully regulate iron transport across the blood-brain barrier the first 6 months after birth, it is paramount that infants have adequate iron stores at birth.

**Figure 1:**
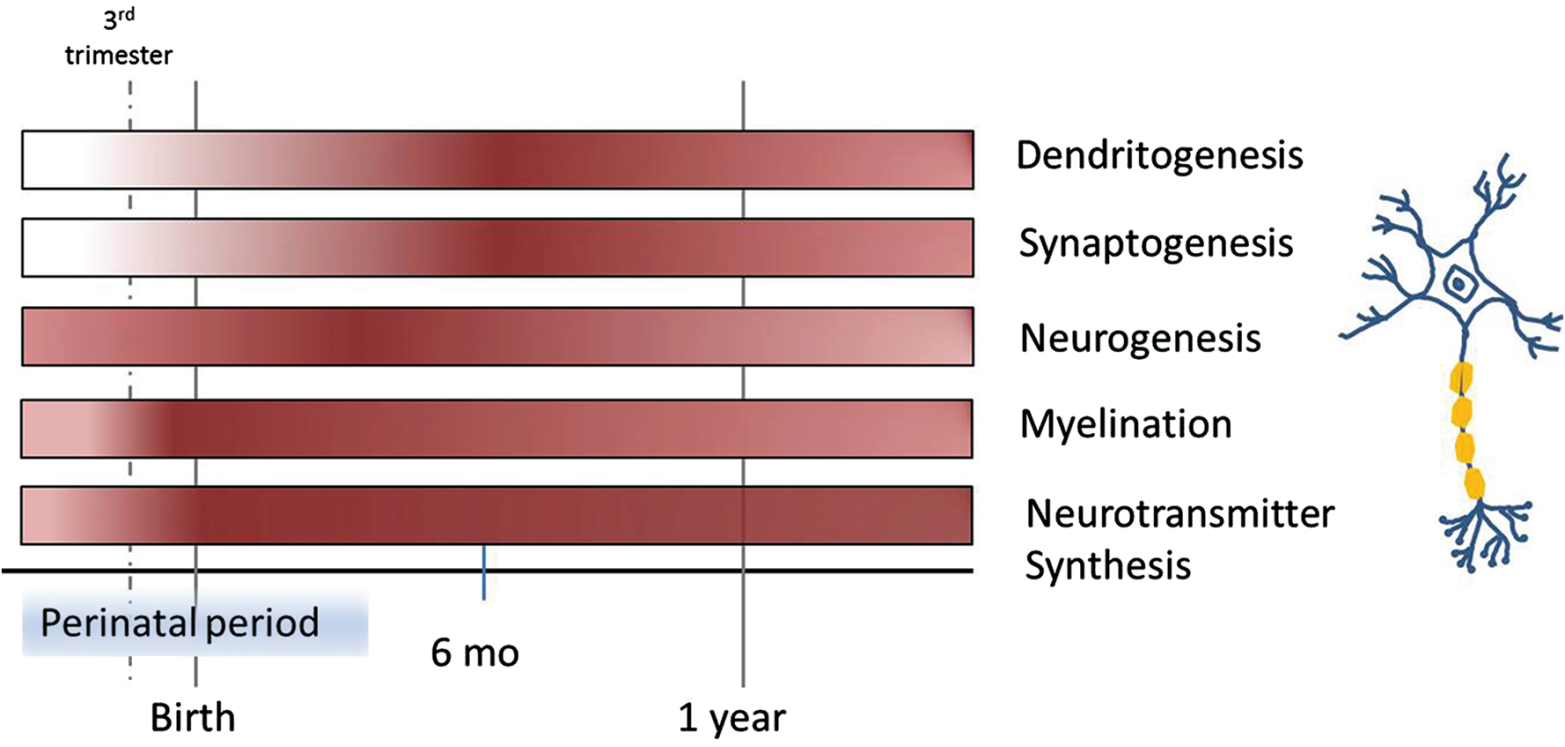
**Time course of different processes in brain development involved in learning and memory over the first year of life, highlighting periods of vulnerability to iron deficiency**. Color intensity corresponds with the start, as well as peaks within each process.

Although hippocampal neurogenesis continues into adulthood, it occurs at a much greater rate prenatally and in the early postnatal period (Figure 1). The newborn neurons integrate into the developing neural circuitry and are thought to be important for learning and memory. Thus, in critical developmental periods environmental insults that inhibit neurogenesis or alter neuron maturation will likely affect present and future behavior. Iron deficiency has been shown to inhibit neurogenesis in the developing hippocampus. Young rats whose mothers were fed an iron-deficient diet and who were born with iron deficiency, showed decreased brain-derived neurotrophic factor (BDNF) levels, down-regulation of BDNF target genes, and altered neuronal differentiation (Tran et al., 2008). Another study was carried out to investigate the effects of perinatal iron deficiency on hippocampal development in mice (Ranade et al., 2013). Iron deficiency in the pre- or postnatal period reduced BDNF and neurogenesis in the hippocampal dentate gyrus of pups. The impact of iron deficiency persisted, as the numbers of hippocampal pyramidal and granule cells were reduced in adults. Moreover, the structural and molecular defects in the pups were correlated with performance in hippocampal-dependent behavioral tasks, with pups from dams that were iron deficient throughout pregnancy and lactation displaying a broad array of defects, while pups from dams that were iron deficient only during pregnancy or during lactation displaying subsets of defects. These findings suggest that iron homeostasis is critical for the expression of neurotrophic factors that support brain development and provide a molecular basis for behavioral deficits related to perinatal iron deficiency (Lozoff et al., 2006).

Iron deficiency can cause changes to neuronal morphology. Neuronal shape is directly related to the computations performed by the cell and is crucial to information processing. Two key morphological features of neurons are dendritic arbor structure and spine density and geometry (Spruston, 2008). The developing arbor requires external inputs to stimulate and support branching morphogenesis. The arbor relies on external cues BDNF that signal via their respective transmembrane proteins. These in turn modulate factors that facilitate elongation and branching by promoting or reducing actin polymerization (Ackermann and Matus, 2003; Sekino et al., 2007). This process is not restricted to early developmental periods because it is also necessary for reshaping neural circuitry during experience-dependent learning throughout life (i.e., synaptic plasticity; Figure 1; Bagot et al., 2009). Like dendritic arbor structure, a spine’s morphology can impact its function (Spruston, 2008). At post-natal day 15, CA1 pyramidal neurons in rats that underwent a period of iron deficiency *in utero* showed reduced dendritic branching and smaller spine head diameters (Brunette et al., 2010). Smaller spine heads could reduce conduction velocity resulting in less coordinated input to the soma as well as represent smaller post-synaptic density which could also affect signal transmission (Hodgkin, 1954).

Consistent with the structural changes noted above, iron deficiency also impacts synaptic plasticity in the developing hippocampus of rats (Jorgenson et al., 2005). For example, prenatal iron deficiency disrupted synaptic plasticity in the developing CA1 region of the hippocampus, but these differences were also apparent in adulthood, after iron repletion. When iron was replete, rat pups demonstrated no developmental increase in synaptic strength, as seen in control animals. It is hypothesized that major developmental events related to proper dendrite outgrowth and synaptogenesis were thwarted due to the unavailability of adequate iron (Jorgenson et al., 2005). This certainly may contribute to the lasting effects of iron deficiency on hippocampal structure and function.

In addition to marked changes in dendrite out growth and synaptogenesis, hypomyelination occurs when iron availability is limited. Proper myelination is important for rapid impulse transmission along axons. Myelination begins in the third trimester of the fetal period and progresses rapidly through infancy (Figure 1; Nakagawa et al., 1998). In the central nervous system, oligodendrocytes are responsible for myelination of axons. As mentioned before, oligodendrocytes synthesize Tf to mobilize needed iron, assuming it is readily available. Studies in humans and rats have demonstrated that iron deficiency can severely affect myelination. Increased latency of auditory brain stem potentials and visual evoked potentials (indirect markers of myelination) has been reported in iron deficient children (Roncagliolo et al., 1998; Algarín et al., 2003). Oligodendrocytes synthesize fatty acids and cholesterol for myelin (Mackler et al., 1979; McKay et al., 1983). In a rat model, restriction of dietary iron during gestation and the early post-natal period resulted in significantly less myelin proteins, lipids, and cholesterol in the spinal cord, brain stem, and cerebellar white matter (Yu et al., 1986; Ortiz et al., 2004). Additionally, rats that were iron deficient in the perinatal period displayed deficits in myelinogenesis at adulthood, even though iron stores were replete (Ortiz et al., 2004). Finally, placing rats on an iron-deficient diet post-weaning lead to a significant decrease in myelination indices in the hindbrain and cerebrum (Beard and Connor, 2003). This suggests that the need and usage of iron by oligodendrocytes does not end during the perinatal period and that the adult brain still requires adequate iron.

Iron is also essential for a number of enzymes involved in neurotransmitter synthesis, including tryptophan hydroxylase used to produce serotonin, and tyrosine hydroxylase used to synthesize norepinephrine and dopamine. Neurotransmitter synthesis begins during embryogenesis (Figure 1; Herlenius and Lagercrantz, 2001). Dopamine is important for regulating cognition and emotion, reward and pleasure, movement, and hormone release (Dunnett et al., 2005). Striatal networks, with dopamine as the major neurotransmitter, relate to higher order cognitive and emotional processes, motivated behavior, positive affect, and reward-related processing, as well as motor function (Dunnett et al., 2005). Studies in humans have shown that young adults, who were iron deficient while an infant, tended to perform more poorly on tasks that involved inhibitory control, set-shifting, and planning, all of which are classified as executive functions that utilize striatal networks that rely on dopamine (Lukowski et al., 2010). Studies in rodents have determined that dopaminergic neurons co-localize with iron throughout the brain, that extracellular dopamine and norepinephrine are elevated in brains of iron-deficient rats, and that the density of dopamine receptors are altered in iron deficiency. These alterations are tightly connected to the extent of iron loss in each brain region (Beard and Connor, 2003). Other studies demonstrate that serotonin transporter (SERT) and norepinephrine transporter densities are also altered by iron deficiency (Burhans et al., 2005). Serotonin is particularly important for proper wiring of neural circuits and is highly implicated in neurodevelopmental disorders, such as autism, anxiety, or depression (Calabrese et al., 2013). The SERT, which is responsible for re-uptake of serotonin within the brain, is the predominant mechanism controlling strength and duration of serotonergic neurotransmission (Gaspar et al., 2003). SERT is expressed more during development than in adulthood (Daws and Gould, 2011). Iron deficiency leads to decreased expression of SERT which in turn exacerbates the decreased expression of BDNF. As previously noted, a reduction in BDNF can have serious consequences for hippocampal structure and function leading to deficits in learning and memory.

## Conclusion

Iron deficiency is the leading micronutrient deficiency in the world. It affects people of all ages, but is most detrimental to infants and children. In developing countries, approximately 12% of children under 5 will die from a micronutrient deficiency (Ahmed et al., 2012). Children who survive will likely be iron deficient, if not anemic. In industrialized countries like the United States, the incidence of iron deficiency is increasing, probably due in part to the increase in the number of children and adults who are overweight or obese (inflammation disrupts iron homeostasis) (Del Giudice et al., 2009). As iron deficiency inhibits learning as well as motor and emotional development, individuals exposed to perinatal iron deficiency are at high risk for failing to reach educational milestones later in life. Moreover, as adults they are more likely to bear children who also experience iron deficiency. Hence, iron deficiency in one generation can beget iron deficiency in the next generation, and so on. Given the consequences of developmental delays in neurocognitive function, such as decreased motor development, lower IQ, difficulties with learning and memory (refer to Table 2 for more evidence), strategies to prevent perinatal iron deficiencies and to promote neural plasticity in individuals exposed to poor iron status during key developmental periods, warrants more attention.
